# Highly Pathogenic Clade 2.3.4.4b H5N1 Influenza Virus in Seabirds in France, 2022–2023

**DOI:** 10.1155/tbed/8895883

**Published:** 2025-02-12

**Authors:** Francois-Xavier Briand, Loïc Palumbo, Claire Martenot, Pascale Massin, Martine Cherbonnel, Rachel Busson, Katell Louboutin, Angelina Orosco, Carole Guillemoto, Florent Souchaud, Isabelle Pierre, Edouard Hirchaud, Manon Tasset, Yannick Blanchard, Nolwenn Le Moal, Anne Van De Wiele, Audrey Schmitz, Eric Niqueux, Béatrice Grasland

**Affiliations:** ^1^Ploufragan-Plouzané-Niort Laboratory, Anses, Ploufragan, France; ^2^French Biodiversity Agency (OFB), Vincennes, France; ^3^Office of Animal Health, French General Directorate for Food (DGAl), Paris, France

**Keywords:** genotype, H5N1 subtype, highly pathogenic influenza A virus, seabirds

## Abstract

In 2022, a very high number of wild bird deaths associated with the detection of highly pathogenic (HP) H5 avian influenza virus (AIV) lineage Gs/GD/96, clade 2.3.4.4b viruses were unusually observed in Europe between May and September, whereas prior to 2022 most of these HP H5 AIVs detected in wild birds in Europe were almost all detected between October and March and few between April and September. In France, wild birds affected by this virus during this unusual period were mainly seabirds, including larids and sulids. Although the abnormal mortalities in larids and sulids were reported simultaneously, sequencing of the complete genomes of the viruses identified in these seabirds showed that sulids are mainly infected with genotype EA-2020-C, whereas larids are mainly infected with genotype EA-2022-BB. The identification of these two genotypes, therefore, confirmed that there was no direct link between the abnormal mortality observed in sulids and the abnormal mortality observed in larids. These two seabird mortality events can also be distinguished by the evolutionary pattern of the number of detections. Indeed, sulid mortality associated with the EA-2020-C genotype was observed in France only between July and September, corresponding to a single epidemic wave, whereas larid mortality associated with the EA-2022-BB genotype began in France and Europe in May 2022 and then this genotype continued to spread among larids in France in the form of several successive epidemic waves until at least September 2023.

## 1. Introduction

Influenza A viruses are enveloped viruses of the *Alphainfluenzavirus* genus in the Orthomyxoviridae family. Their negative-stranded RNA genome consists of eight segments encoding a total of 10–14 proteins. Avian influenza viruses (AIVs) are classified on the basis of antigenic differences in their surface glycoproteins, hemagglutinin (H1–H16), and neuraminidase (N1–N9) [1] and was also suggested an additional H19 subtype [2]. The genetic evolution of AIVs is driven by two main events, which induced a very high genetic diversity of these viruses. The first one corresponds to an evolution by mutations (substitutions, insertions, and deletions) and the second one corresponds to an evolution by reassortment events [1]. The reassortment events occur when the same host has been coinfected by two influenza viruses. Since 1996, highly pathogenic (HP) H5 viruses of the A/goose/Guangdong/1/96 (Gs/GD/96) lineage have caused recurrent outbreaks with high mortality in both wild and domestic birds. These HP AIVs of this lineage are classified into 10 different clades (0–9) and subclades based on hemagglutinin sequences [3]. This lineage first spread in Asia, before becoming more common in Europe and Africa each winter and has even spreading massively to North and South America since late 2021 [4–6]. For the last 4 years, Europe has been facing massive outbreaks every year specifically with the H5 of clade 2.3.4.4b [7] until it seems to be endemic in Europe [4]. In 2022, a very large number of wild bird deaths associated with the detection of HP H5 AIVs were unusually observed between May and September, while before 2022 HP H5 AIVs detected in Europe were almost all detected between October and March and few between April and September. The wild birds affected by this virus were mainly seabirds, including larids and sulids [7].

A wide genetic diversity of these viruses has been observed in Europe due to many reassortment events between the H5 2.3.4.4b clade and other low pathogenic viruses in wild birds. From 2020 to 2022 in Europe, at least 50 new HP H5 genotypes were observed, showing the huge diversity of these viruses [8]. Amongst these genotypes, the genotype EA-2020-A (H5N8) was largely predominant during the epizootic period 2020–2021 in Europe, whereas during the next epizootic period 2021–2022, the genotype EA-2020-C H5N1 was initially in majority before being replaced by the EA-2021-AB (H5N1). Before the epizootic 2022, no specific study based on epidemiological or genetic data was reported on avian influenza in sulids. In contrast, many studies on avian influenza in gulls have been reported [9–11]. In particular, the H13 and H16 AIV subtypes are predominant in gull populations, but represent only a small fraction of AIVs detected in other avian families, such as Anatidae. In addition, the genetic analysis of the eight segments of H13 and H16 viruses differs significantly from that of other AIVs circulating in different wild bird hosts [11, 12]. Moreover, experimental infections with this subtype demonstrated that the infection does not readily occur on ducks [13, 14] which could be explained by the receptor-binding properties of H13 and H16 viruses [15]. Black-headed gulls (*Chroicocephalus ridibundus*) are gregarious birds present inland during winter (lakes, ponds, swamps, etc.), while along coastlines for nesting. Populations are broadly distributed in Europe, encountered in urban areas as well as in wetlands, with thousands of individuals that might be found in the same area. They are either partial migrants (especially the southern populations), which do not follow a common route during the breeding season or migratory (northern and eastern populations), which can travel long distances. In Western Europe, including France, wintering and nesting areas overlap, therefore, gulls can be observed all year long in wetlands and coastal sites. Moreover, France occupies a central place in migratory schemes. In France, during winter individuals are mainly found in a northwest quarter of the country, while they can be found in all territory during the nesting season [16].

Herring gulls (*Larus argentatus*) are opportunistic feeders, that might feed in Sea as well as in urban areas close to human activity (e.g., landfills). In France, they are mainly located on coastline, breeding in colonies—from the Belgium boarder to the southwest Atlantic coast. Nonetheless individuals can be encountered inland along main rivers, including in cities. The two subspecies are partial migrants but with distinct schemes. While populations of *L. argentatus argentatus* overwinter mainly in northern and western Europe (Northern Sea, Baltic Sea, and English Channel), populations of *L. argentatus argenteus* are mainly non-migratory with short-distance dispersal and no common oriented trajectory around colonies (although juveniles may undertake long-distance dispersal along the coastline) [16].

Northern Gannets (*Morus bassanus*) are piscivorous birds, nesting in colonies (close nests) in western regions (especially Scotland) and East Canada with important fidelity to their nesting site in cliffs. In France, most breeding individuals nest in the colony “des Sept-Iles” in Britany, present from February to late summer [17]. This population, like some other European populations, winters in southern regions (Mediterranean or North Africa) for individuals breeding in southern colonies. Populations breeding in northern Europe, wintering in northern and western Europe (Northern Sea, Baltic Sea, English Channel). Solitary individuals or small groups can be encountered in France during migration and winter periods, although most migration routes are pelagic [16].

This study, therefore, focuses on these unusual detections of HP H5 AIVs in seabirds, characterizing the geographical and temporal distribution, and the species affected by these viruses in France using phylogenetic analyses.

## 2. Materials and Methods

### 2.1. Detection of HP H5 AIVs From Wild Birds

From May 2022 to August 2023 in France, for each suspected case of HP AIVs in wild birds, oropharyngeal, and cloacal swabs were collected only from deceased birds by the SAGIR network (a French wildlife health surveillance network composed of the French Biodiversity Agency, hunters, and veterinary laboratories). Swab supernatants could be pooled by a set of five if they were from the same species, collection site, and date (except for samples from swans, which were analyzed individually). Viral RNAs were extracted from swab supernatants and then tested by rRT-PCR targeting the M gene and H5 gene [18] by approved screening laboratories. The positive samples were then transferred to the National Reference Laboratory for Avian Influenza (AI French NRL, Anses-Ploufragan-Plouzané-Niort) for specific rRT-PCR of the H5 gene of clade 2.3.4.4b [19] and the N1 gene [20]. The RNA with earliest *Cq* value for rRT-PCR H5 in the different RNAs obtained from the same wild species, same location in France, and same date was selected to obtain a full genome viral sequence. The eight viral segments were amplified by RT-PCR [21], then preparation of the libraries were performed followed by the deep-sequencing on an IonProton sequencer (life technologies) as already described [22]. Only samples with almost fully sequenced genomic segments (>95% of complete genome length available) were used for the following analyses and were deposited to GISAID database (Supporting Information 12: Table S1).

### 2.2. Phylogenetic Analyses and Genotype Determination

Each obtained viral segment was compared by BLAST with the available sequences in international databases (GISAID; data retrieved in week 48, 2024). For the earliest French viral sequences of genotypes EA-2020-C and EA-2022-BB detected in France, the 200 closest sequences were retrieved and added with the French sequences to constitute a set of sequences for each segment. Redundant sequences and sequences exhibited too poor quality (too many ambiguous nucleotides positions) were deleted. Reference viruses of genotype EA2022-BB, genotype EA-2020-C, and genotype EA-2021-AB were also added of the set of sequences. Segment sequences were aligned using the MAFTT v7 software [23]. The phylogenetic analysis was also performed with IQ-TREE v2.3.5 with using the best-fitted nucleotide substitution model determined by ModelFinder and 1000 ultrafast bootstrap replicates [24, 25]. Based on these results, the genotypes of the French viruses were determined according the phylogenetic clustering of their eight segments. The name of each genotype detected in France was given according the proposed unified nomenclature described by Fusaro et al. [8]. For intragenotype comparison, the eight viral segments were concatenated to optimize the difference between the genomes of viruses. Then the phylogenetic analysis was performed as above.

### 2.3. Emergence/Introduction and Spatiotemporal Spread Analyses

First, for the major genotype detected from larids, a spatiotemporal representation was performed according the collection date and geolocation of each selected sequenced sample. Second, spatiotemporal analyses based on the concatenated genome were performed using BEAST v1.8 [26] in association with beagle library [27]. The best-fit nucleotide substitution model was selected using Modeltest [28]. A general time reverse (GTR) substitution model with gamma distribution (+G4) as nucleotide substitution model, uncorrelated lognormal relaxed molecular clock model and the coalescent constant population size model were used [29]. The collection dates were included in the analyses as well as the geolocation of each sample as continuous traits. A Cauchy relaxed random walk model of evolution was chosen to accommodate differences in diffusion rates across branches [30]. The Markov Monte Carlo chains (MCMCs) were run between 200 million iterations and sampled every 20,000 with 10% as for segment burning. To visualize the enough mixing and convergence properties the parameters obtained were with Tracer v1.6 (all parameters were >200). The maximum clade credibility (MCC) trees were summarized with TreeAnnotator v1.8 software and visualized with Figtree v1.4.2 software. The patterns of dispersion were visualized by Evolaps2 [31]. According to the results obtained, five geographical areas were defined within Evolaps2, allowing the continuous parameter of location to be summarized in a discrete parameter.

In order to estimate the date of emergence of this larids genotype, or at least the date of introduction of this genotype into France, the time to most recent common ancestor (tMRCA) was calculated on the basis of the HA segment as well as on the basis of the concatenated segments that were common between this genotype and parental HP AIV genotype. So French viruses of this genotype detected from May 2022 to September 2022 and the sequences from Europe, Asia, and Africa from September 2021 to October 2023 belonging to the parental HP H5 AIV genotype. A maximum likelihood tree with 1000 bootstrap replicates was constructed with IQ-tree [32] to check for the presence of temporal signal by TempEST v1.5 [33]. To improve the temporal signals in our dataset, all outliers were identified and removed. The linear regression of the genetic distance from root-to-tip against the time of sampling using residual mean square (RMS) showed a temporal signal with *R*^2^ = 0.82. Subsequently, as in previous analyses, the GTR + G substitution model with gamma distribution (+G4) nucleotide substitution model, the uncorrelated lognormal relaxed molecular clock model were used and three coalescent population size models were compared (skygrid, constant, and exponential) [19].

## 3. Results

### 3.1. Detection and Geographic Distribution

From May 2022 to August 2023, cloacal and oropharyngeal swabs were collected from 2865 dead wild birds to detect AIVs. This number is higher than in previous years, as from May 2020 to August 2021 with 595 dead wild birds collected or as from May 2021 to August 2022 with 1340 dead wild birds collected. Among the 2865 wild birds, the 632 complete genomes of HP H5 AIVs were obtained. Among these genomes, 466 viral genomes were identified from seabirds representing 74% of the complete genomes obtained from wild birds in France. In this study, seabirds include only the bird families: sulids (Northern Gannets) and larids (gulls and terns). Of these 466 genomes, 36 were identified from sulids and 430 from larids. The number of detections and viral sequences obtained only reflects a glimpse of the real number of dead birds linked to AIVs, as in principle only a maximum of one sample was taken per week and per municipality for each dead species. Furthermore, in the case of pelagic birds, some may have died on the high seas, and therefore, been uncounted and harvested. The total accounts have been made of reported mortalities (results not available), but this remains a large underestimation of actual mortalities and remains difficult to assess.

**Figure 1:**
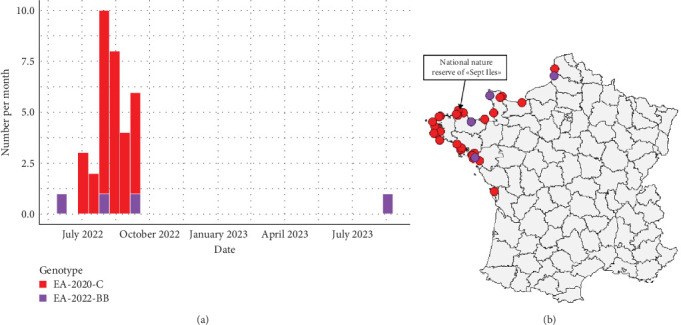
Temporal repartition of the number of each highly pathogenic (HP) H5 avian influenza virus (AIV) clade 2.3.4.4b genotype identified from Northern Gannet in France from May 2022 to August 2023 (a). Geographical location of the genotype identified from Northern Gannet in France from May 2022 to August 2023 (b).

The comparison of the eight phylogenetic trees of each viral segment (Figures S2–S10) indicates that two genotypes were identified from sulids in France. The genotype EA-2020-C was detected 32 times, whereas the genotype EA-2022-BB was identified only four times. Detection of the genotype EA-2022-BB in Northern Gannets appears to be sporadic throughout the studied period (Figure 1a). In contrast, the genotype EA-2020-C appears to be a unique big wave of detection between July and September 2022. This EA-2020-C genotype was only detected in gannets during the period studied. The first cases of the EA-2020-C were detected near France's largest gannet colony in northern Brittany (Figure 1b). Subsequent cases were mainly detected on the northwest coast of France. Finally, the last cases were the most southerly along the Atlantic coast.

**Figure 2:**
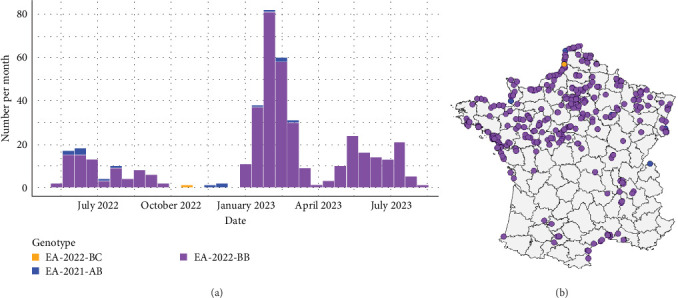
Temporal repartition of the number of each highly pathogenic (HP) H5 avian influenza virus (AIV) clade 2.3.4.4b genotype identified from gulls in France from May 2022 to August 2023 (a). Geographical location of the genotype identified from gulls in France from May 2022 to August 2023 (b).

For the larids, on the same studied period, three genotypes were detected in France. One virus was to genotype EA-2022-BC, 15 virus belonged to the genotype EA-2021-AB and 414 were genotype EA-2022-BB (Figure 2a). The genotype EA-2021-AB was detected sporadically until February 2023. This genotype has been implicated in high larid mortality in northern Europe as in Netherlands in May–June 2022 [34].

**Figure 3:**
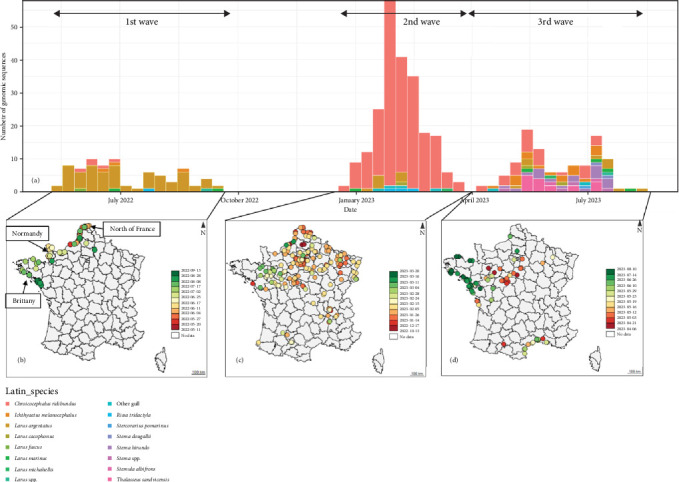
Temporal and species repartition of the number of the genotype EA-2022-BB identified from larids in France from May 2022 to August 2023 (a). Temporal and geographic repartition of the genotype EA-2022-BB during the first wave (b), the second wave (c), and the third wave (d).

For the genotype EA-2022-BB, the number of detection seems to be distributed through three major epizootic waves (Figure 2a,b). The first wave occurred from mid-May 2022 to mid-September 2022. During this wave, genotype EA-2022-BB predominantly affected European Herring gulls (Figure 3), which accounted for 73.2% of genotyped viruses. Their geolocations were clearly along the coastline in the northern part of the France. The spatiotemporal analysis of this wave based on the collection dates shows a first detection in the north of France close to the Belgian border, then the frontline of detected cases moved southern-westwards to Normandy and Brittany to reach the south of Brittany at the end of August. After this first epizootic wave of genotype EA-2022-BB, from September 2022 to end of December 2022 no genome belong to this genotype has been detected in France.

Then, the second epizootic wave was observed from the end of December 2022 to March 2023 with 244 virus obtained sequences from larids. In contrast to the first outbreak the vast majority of cases (91%) coming from black-headed gulls (Figure 3). The locations of the cases were no longer along the coastline as the first wave, but mainly inner land of the northern part of France (Figure 3). The first cases were detected in Northern France. Thereafter, many cases were rapidly observed in the Paris region and in the northeastern quarter of France. Many cases were also detected nearby the Rhône River. The end of this wave was noticeable with an increase of detection of this genotype in northwestern part of France. Few cases were also observed in southwestern part of France along the Garonne River. The spatiotemporal analysis of this wave is more complex than that of the first wave. It is highly likely that the EA-2022-BB viruses detected in France during this second wave originated from multiple introductions into French territory following the spread of the viruses in other countries (Supporting Information 11: Figure S11), adding to the viruses of this genotype already circulating in France with low noise. It would, therefore, be necessary to carry out, with all countries concerned, global phylogeographic, and phylodynamic analyses integrating as many sequences as possible.

Finally, the third epizootic wave of genotype EA-2022-BB was observed in France from April 2023 to August 2023. During this wave, 66 viral sequences of this genotype were identified from larids. The spatial distribution and species distribution were more heterogeneous than the two previous waves, with detection from black-headed-gulls mainly in inner land of northwestern part of France and terns mainly in sea in western coastline of France and in Mediterranean coastline, respectively (Figure 3).

### 3.2. Intragenotypic Phylogenetic Analyses of the Major Genotypes Detected in Seabirds

**Figure 4:**
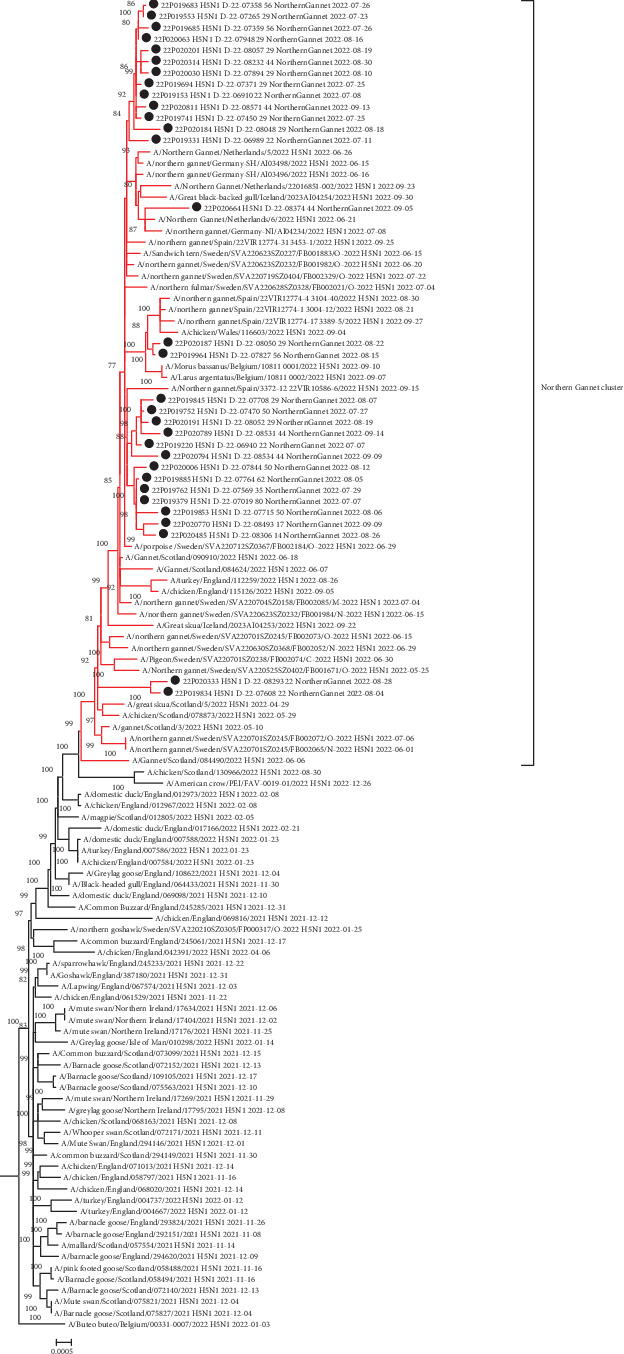
Phylogenetics tree based on the eight concatenated segments of avian influenza virus (AIV) highly pathogenic (HP) H5 AIVs detected from Northern Gannet in France and their closest related sequences belong to the genotype EA-2021-C. Subtree in red representing a specific phylogenetic cluster of virus identified from Northern Gannets in Europe. The French sequences were indicated by a black dot. Only bootstrap values above 75 as branch supports were indicated.

The genotype EA-2020-C is the most frequent genotype found in Northern Gannets during the studied period in France and is also one of the two most frequently detected genotypes during the 2021–2022 epizootic period in Europe and in France, in both domestic and wild birds. Phylogenetic analysis of the eight independent segments and concatenated genomes showed that the viral sequences detected in France in Gannets formed a monophyletic cluster with the other viruses detected in Gannets in northern Europe and Spain between May 2022 and October 2022 (Figure 4). Within the specific cluster of Gannets, several subclusters including French sequences were observed, suggesting different introductions of this virus into the French Gannet population.

EA-2022-BB was first detected in northern France on May 11, 2022, from a European Herring Gull, marking its initial appearance. Based on the phylogenetic analyses of each segment, the genotype seems to be a reassortant virus between genotype EA-2021-AB and very likely with a virus H13 which corresponds to a subtype of viruses that predominantly infected larids. According the phylogenetic analyses, the viral segment of the genotype EA-2022-BB was composed by PB2/PB1/HA/NA/M segments from a genotype EA-2021-AB while segments PA/NP/NS were from a H13Nx viruses (Supporting Information 2: Figure S2).

### 3.3. Viral Spread of Genotype EA-2022-BB During the First Wave

**Figure 5:**
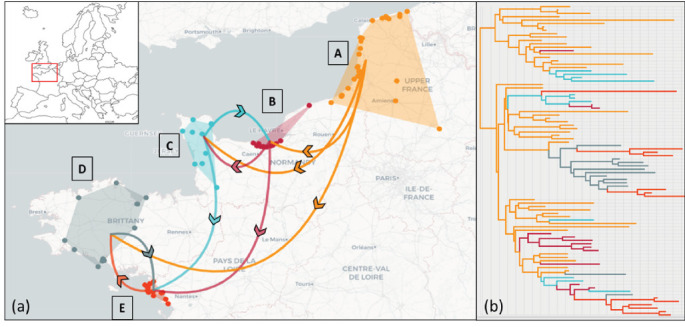
Schematic viral spread based on concatenated sequences of the genotype EA-2022-BB during the first wave from larids in France (a). Maximum clade credibility (MCC) tree based on concatenated sequences of the genotype EA-2022-BB during the first wave in France (b). Each branch color corresponds to the geographic area.

The estimated origin of the genotype EA-2022-BB in France came from the Northern France (Area A) near bay of Somme's nature ornithological reserve. In this area, the virus was detected throughout the period studied. From this Area A, the virus seems to spread directly to Area B, C, and D. According to the results obtained, this genotype has spread directly from A to B area at least three times (Figure 5). In similar way, the virus has directly spread from Area A to Area C at least four times. The viral spread from area A to area D was estimated to two times. The first viral spread from A to D area led to a large expansion inside this new affected area, whereas the second viral spread from Area A to D led to an only one sporadic detection. The area E has been affected by the virus from the area B, C, and D (two times from D area and one time from B and C areas; Figure 5; Supprting Information 1: Figure S1).

Based on the concatenation of segments, the determination of time to common ancestor (tMRCA) of all French genotype EA-2022-BB of the first wave was estimated to be during the second half of March 2022. With an early estimated mean was for the exponential coalescent model (mean tMRCA 18 March 2022; 95% HPD between 1 March to 4 April), followed by the constant coalescent model [21] (March; 95% HPD between 3 March to 8 April) and the late was the skygrid coalescent model [29] (March 2022; 95% HPD between 9 March to 16 April). These results suggest that there was just over a month between the common ancestor of the French sequences and the first detection (11 May, 2022). The results of analyses based only on HA sequences give similar results, with median tMRCA values ranging from 23 to 26 March, regardless of the coalescent model used, but with wider confidence intervals than the results obtained with the concatenated segments.

## 4. Discussion

While the detection of HP H5 clade 2.3.4.4b AIV cases in Europe usually happened over the winter months (October–March) in both wild and domestic birds, the months of April–September 2022 were characterized by very high mortality rate in wild birds in Europe (almost exclusively seabirds) linked to these viruses. During this period, France was particularly affected by mortality of Northern Gannets and European herring gulls. Although the abnormal mortalities from gulls and Northern Gannets began to be reported concomitantly, sequencing of the complete genomes of the viruses identified in these seabirds indicated that Northern Gannets were mainly infected with genotype EA-2020-C, whereas larids were mainly infected by genotype EA-2022-BB. The identification of these two genotypes, therefore, confirmed that there is no direct link between the abnormal mortality observed in Northern Gannets and the abnormal mortality observed in larids. These two seabird mortality events can also be distinguished for the evolution pattern of the number of detections. Indeed, Northern Gannet mortality associated with the EA-2020-C genotype was in France observed only between July and September, corresponding to a single epidemic wave, whereas larid mortality associated with the EA-2022-BB genotype began in France and Europe in May 2022 and then this genotype continued to spread among larids in France in the form of several successive epidemic waves until at least September 2023.

The genotype EA-2020-C was one of the two most frequently detected genotypes in Europe between late 2020 and early 2022, whether in domestic or wild birds [8]. Prior to the first detection of this genotype in France in Northern Gannets in France, many cases had also been detected in Gannet colonies in the northern British Isles a few weeks earlier [35, 36]. The phylogenetic analyses based on this genotype collected in France and the rest of Europe showed that all these sequences formed a monophyletic cluster. It is, therefore, likely that there was only one introduction of the virus into one of the European Gannet colonies and that the virus spread between the colonies around the North Sea and the English Channel. Moreover, detections were also observed on the coasts of the Iberian peninsula between August and September, probably linked to their migration. This intial introduction probably took place at the start of the nesting season in Europe, when Northern Gannets are aggregated in colonies with very high bird densities, corresponding to extremely favorable conditions for a very fast viral spread into the affected colonies. During the Gannet breeding season, bird movements were generally focused on specific nesting areas with very little overlap between distribution areas. This should have limited the spread of the virus across the different European Gannet colonies. Nevertheless, the identification of several phylogenetic subclusters of HP H5 AIVs in France seems to indicate several exchanges between colonies in Europe during the epidemic wave in France. This observation was supported by the observation of GPS data from several individuals, which showed a change in behavior during the 2022 HP H5 AIV epidemic, with an increase in intercolony exchanges [37]. This change in behavior is likely to have amplified the spread of the virus through the various Gannet colonies, resulting in a large number of wild bird deaths for this particular species. An international comparative study, at inter- and intracontinental level, between the ornithological data and the genomic viral data should be engaged to better understand and try to anticipate the consequences of the viral spread in Gannet populations. The epidemic wave of the genotype EA-2020-C associated with Northern Gannet deaths seems to correspond to the end of detection of this genotype in Europe whether in wild and domestic birds [8].

For the genotype EA-2022-BB observed in the larids, phylogenetic, and phylodynamic analyses suggest that it originated from the reassortment event between the EA-2021-AB genotype and a specific AIV already adapted to larids (probably H13; Supprting Information 2: Figure S2). Given the data available, estimating the age of the common ancestor from the sequences of the first wave suggests an emergence approximately 1 month before the first detection in France, as well as in Europe (in May 2022), underlining the good efficiency of the wild bird surveillance system. It is very likely that a reassortment between the EA-2021-AB genotype and an H13 virus occurred in larids in a geographical area close to the north of France. The EA-2021-AB genotype was the main genotype circulating in Europe at the beginning of 2022 [8]. In particular, it was detected in larids in France between late March and early April 2022. It was probably responsible for tern mortality in northern France and northern Europe in May and June [34], indicating active circulation of this genotype in larids in this region of Europe [38].

Subsequently, spatial and temporal analysis of the first wave, based on geolocation and the date of detection, suggests that the virus spread began in northern France and reached Normandy, then northern Brittany and finally southern Brittany, following the coastline. The incorporation of viral sequence data into the spatiotemporal analyses seems to show that the spread of the virus in France in the first few months was not as straightforward and linear as suggested by the analyze only based on the collection date. The virus arrived in Brittany either via a stopover in Normandy or directly from northern France to Brittany (with a potential jump of almost 450 km in just a few days). Despite a very high apparent prevalence with 414 detection for larids on the period May 2022–September 2023 in France only few other wild birds were affected by this genotype (*n* = 27). The other wild species affected by this genotype were mainly birds of prey (*n* = 15) as peregrin falcons. These birds of preys were probably infected by this genotype by hunting or scavenging on infected larids. In additon, on the study period, only six detections of this genotype were confirmed from domestic birds (five from chicken backyards and one from turkey farm). The poultry farms affected by this virus were located in the same area as affected larids. The high detection rate of this genotype in larids could be explained by two major factors. The first is an environmental factor. In fact, the location and period during which the first wave was observed, which mainly affected herring gulls, corresponds to the breeding season of herring gulls, when these birds gather to nest along the channel and the Atlantic coastline. The second wave, which mainly affected black-headed gulls, also coincided in location and timing with the breeding season of black-headed gulls. The breeding season is an opportune time for birds to be in close proximity to each other, facilitating the spread of the virus in the affected bird population.

The second factor corresponds to an intrinsic characteristic of the virus, which is well adapted to birds in the larids group. The acquisition of the three segments (PA, NP, and NS) from a virus already adapted to gulls (probably H13) by an HP H5 AIV very probably confers a selective advantage for better spread between larids. This hypothesis is supported by the fact that several species of larids have been affected, while other wild bird species have only been sporadically affected. In addition, the genotype EA-2021-AB also infected gulls on several occasions during the same periods as the genotype EA-2022-BB, but without ever-involving massive detection and mortality among the affected larids. The continued circulation of genotype EA-2022-BB viruses during at least 15 months in the larids could increase the adaptation of this virus to these birds, particularly by imposing selection pressure on the PB2 PB1 HA NA M segments, which initially do not come from a specific viral genetic lineage in the larids, as also already observed for the H13 and H16 viruses [39–41]. From September 2023 to February 2024, only six cases of this genotype have been detected in gulls in France, suggesting a persistent but low-noise virus circulation in larids populations. The disappearance of observations of high mortality rates and the decrease in the number of detections of HP H5 AIV in these bird populations could be explained by a drastic reduction in the number of larids susceptible and immunologically naive to this virus. Since the first detection of the genotype EA-2022-BB in France in May 2022, the genotype has subsequently spread widely to most European countries [42–45] and as far East as Russia and Kazakhstan [46]. The genotype has also spread southwards by infecting wild birds in West Africa suggesting transcontinental exchange [42] probably linked to the migration of certain wild birds. This spread was certainly related to the location and intra- and intercontinental movements of the various larid species affected. Moreover, this genotype (EA-2022-BB) has been detected on several occasions in wild mammals such as red foxes (notably in France), but also in domestic mammals raised for fur in several european contries. The first detection in domestic mammals was occurred in a mink farm in Spain in October 2022. The viral spread observed in this mink farm suggests a possible inter mammals transmission of a HP H5 clade 2.3.4.4b AIVs [47]. Since then, the EA-2022-BB HPAIV genotype has also been identified in at least 26 fur farms in Finland, affecting several mammalian species such as American mink, red fox, Arctic fox, and raccoon dog. In these farms, the most likely source of virus introduction was contact with gulls. As previously described, almost all the HP H5 AIVs of the genotype EA-2022-BB showed mutations Y52N on NP [48] and NA-S369I [49], which may increase their zoonotic potential [50]. Despite the very clear host specificity observed in larids, this genotype is and must be particularly under surveillance.

## 5. Conclusion

Over the past 4 years, Europe has been grappling with many outbreaks of avian influenza HP H5 AIVs, resulting in significant fatalities among wild birds and devastating incursions of the virus into farms. Although initially based on similar observations in seabirds (larids and sulids), such as abnormal mortality rates during unusual periods, a 15-month study of HP H5 AIVs in France showed that the characteristics of infected birds, combined with the phenotypic characteristics of the virus, could influence the persistence and spread of the virus within bird populations. The extensive genetic diversity observed in HP H5 AIVs across Europe in recent years, combined with the wide variety of birds (and potentially mammals) susceptible to infection, emphasizes the need for exercising great caution when predicting the future spread of HP H5 AIVs belonging to clade 2.3.4.4b.

## Data Availability

The viral sequences that support the findings of this study were deposited in the GISAID database (https://gisaid.org/). Their accession numbers and the related additional data are available in the supporting information 12: Table S1. The sequences will be released when the data or accession number appear in print.

## Supporting Information

Supporting Information 1 Figure S1: Continous phylogeographic reconstruction of the spread of genotype EA-2022-during the first wave in France.

Supporting Information 2 Figure S2: Schematic representation of the likely reassortment event between the genotypes.

Supporting Information 3 Figure S3: Phylogenetic tree based on the PB2 segment using ML method.

Supporting Information 4 Figure S4: Phylogenetic tree based on the PB1 segment using ML method.

Supporting Information 5 Figure S5: Phylogenetic tree based on the PA segment using ML method.

Supporting Information 6 Figure S6: Phylogenetic tree based on the HA segment using ML method.

Supporting Information 7 Figure S7: Phylogenetic tree based on the NP segment using ML method.

Supporting Information 8 Figure S8: Phylogenetic tree based on the NA segment using ML method.

Supporting Information 9 Figure S9: Phylogenetic tree based on the MP segment using ML method.

Supporting Information 10 Figure S10: Phylogenetic tree based on the NS segment using ML method.

Supporting Information 11 Figure S11: Phylogenetic tree based on the concatenated genome of EA-2022-BB using ML method.

Supporting Information 12 Table S1: Related information's about the French viral sequences used in this study.
