# Supplementary material for: Highly Pathogenic Clade 2.3.4.4b H5N1 Influenza Virus in Seabirds in France, 2022–2023
Source: Transbound Emerg Dis. 2025 Feb 12;2025:8895883. doi: 10.1155/tbed/8895883 (PMC12016834; doi:10.1155/tbed/8895883)
Supplement: Supporting Information 1 — Figure S1: Continous phylogeographic reconstruction of the spread of genotype EA-2022-during the first wave in France. [file 8895883.f1.pptx]

## Slide 1
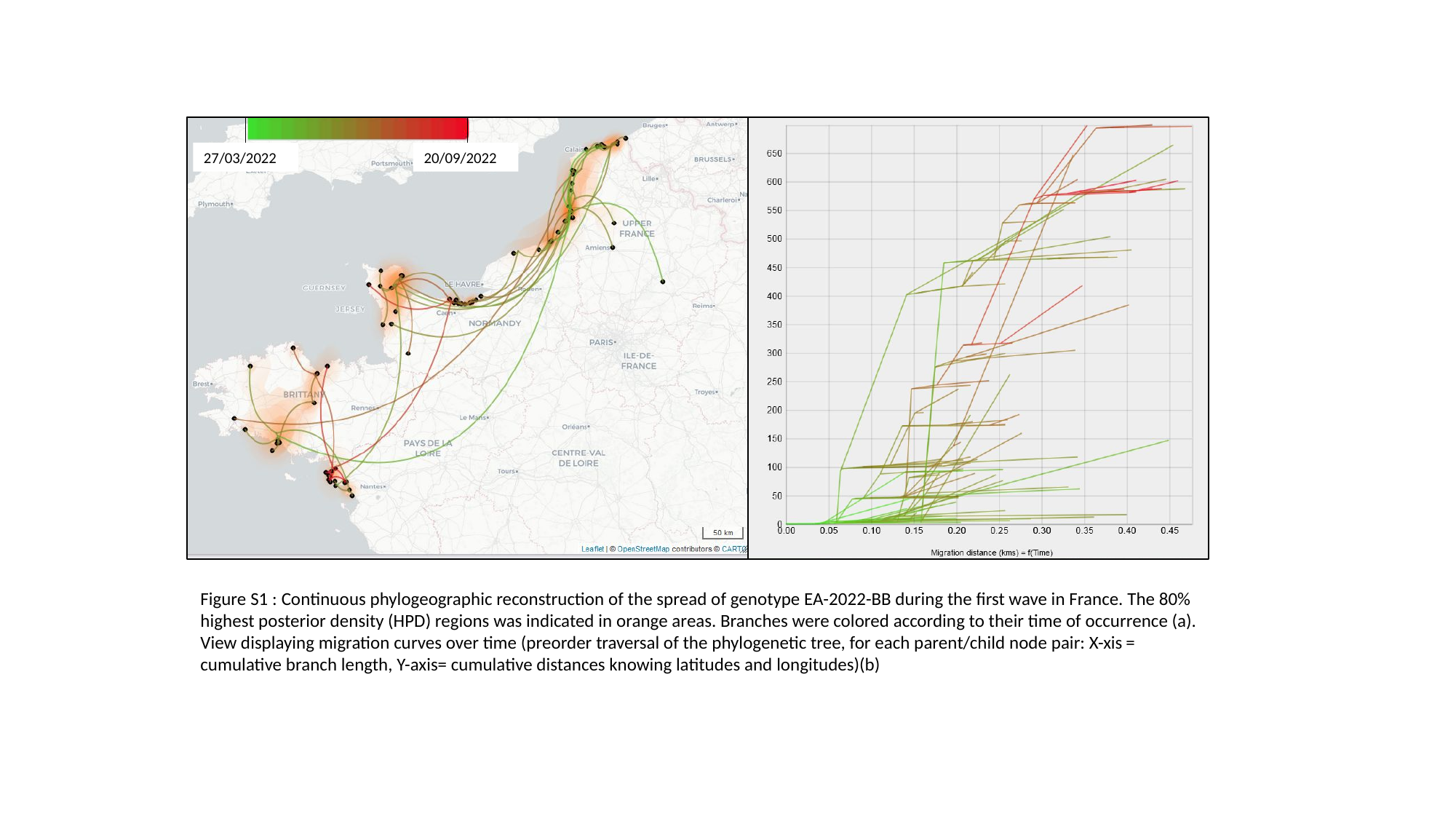

20/09/2022
27/03/2022
Figure S1 : Continuous phylogeographic reconstruction of the spread of genotype EA-2022-BB during the first wave in France. The 80% highest posterior density (HPD) regions was indicated in orange areas. Branches were colored according to their time of occurrence (a). View displaying migration curves over time (preorder traversal of the phylogenetic tree, for each parent/child node pair: X-xis = cumulative branch length, Y-axis= cumulative distances knowing latitudes and longitudes)(b)
