# Supplementary material for: Highly Pathogenic Clade 2.3.4.4b H5N1 Influenza Virus in Seabirds in France, 2022–2023
Source: Transbound Emerg Dis. 2025 Feb 12;2025:8895883. doi: 10.1155/tbed/8895883 (PMC12016834; doi:10.1155/tbed/8895883)
Supplement: Supporting Information 2 — Figure S2: Schematic representation of the likely reassortment event between the genotypes. [file 8895883.f2.pptx]

## Slide 1
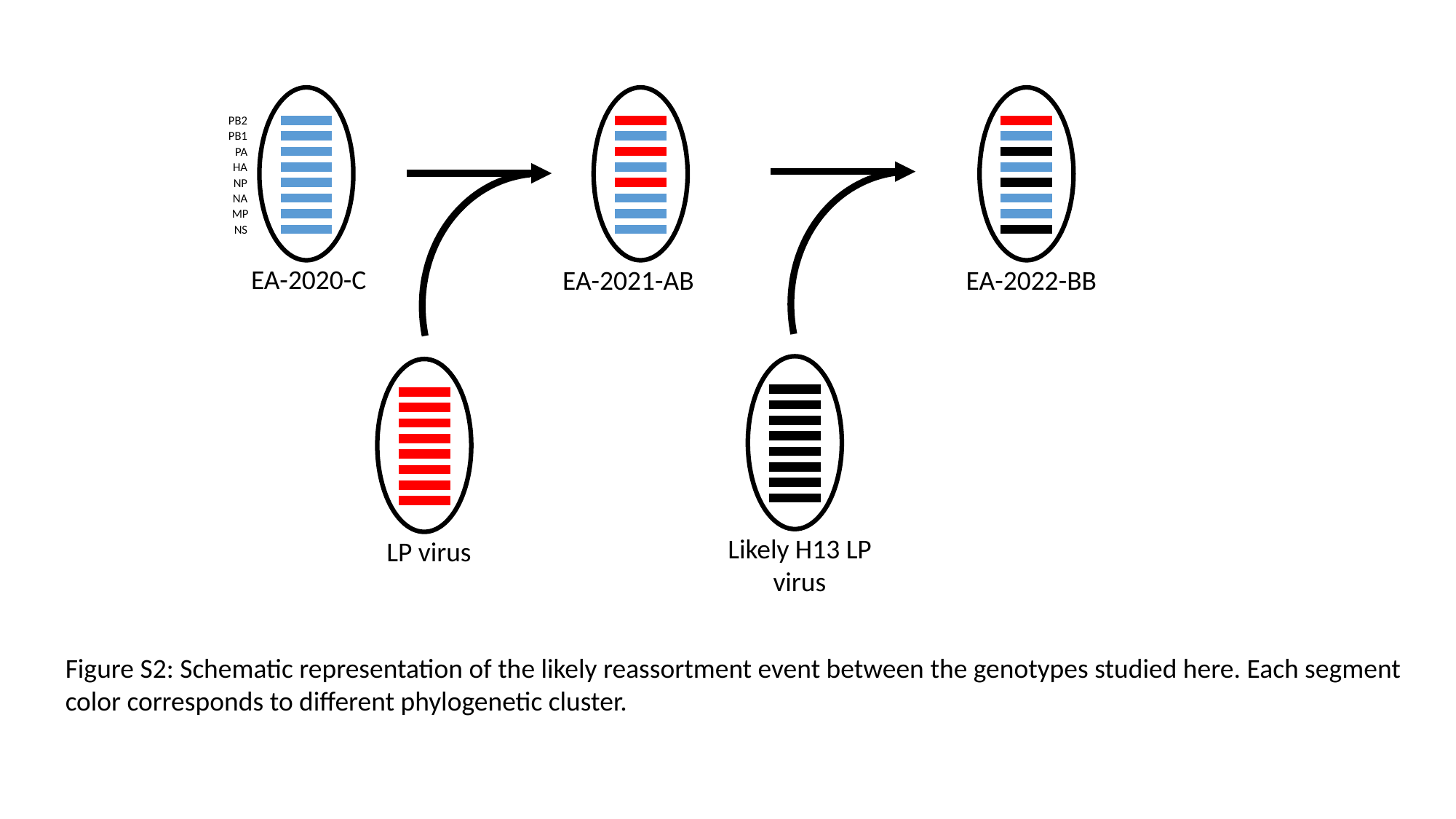

PB2
PB1
PA
HA
NP
NA
MP
NS
EA-2020-C
EA-2021-AB
EA-2022-BB
Likely H13 LP virus
LP virus
Figure S2: Schematic representation of the likely reassortment event between the genotypes studied here. Each segment color corresponds to different phylogenetic cluster.
