# Supplementary material for: Highly Pathogenic Clade 2.3.4.4b H5N1 Influenza Virus in Seabirds in France, 2022–2023
Source: Transbound Emerg Dis. 2025 Feb 12;2025:8895883. doi: 10.1155/tbed/8895883 (PMC12016834; doi:10.1155/tbed/8895883)
Supplement: Supporting Information 10 — Figure S10: Phylogenetic tree based on the NS segment using ML method. [file 8895883.f10.pdf]

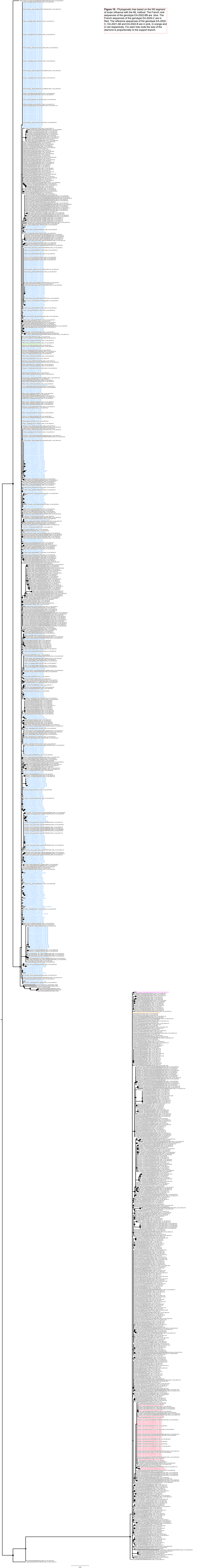

Figure 10 : Phylogenetic tree based on the NS segment of avian influenza with the ML method. The French viral sequences of the genotype EA-2022-BB are blue. The French sequences of the genotype EA-2020-C are in Red. The reference sequences of the genotype EA-2020-C; EA-2021-AB and EA-2022-B are in pink, in orange and in red respectively. For each tree node the size of the diamond is proportionally to the support branch.
