# Supplementary material for: Highly Pathogenic Clade 2.3.4.4b H5N1 Influenza Virus in Seabirds in France, 2022–2023
Source: Transbound Emerg Dis. 2025 Feb 12;2025:8895883. doi: 10.1155/tbed/8895883 (PMC12016834; doi:10.1155/tbed/8895883)

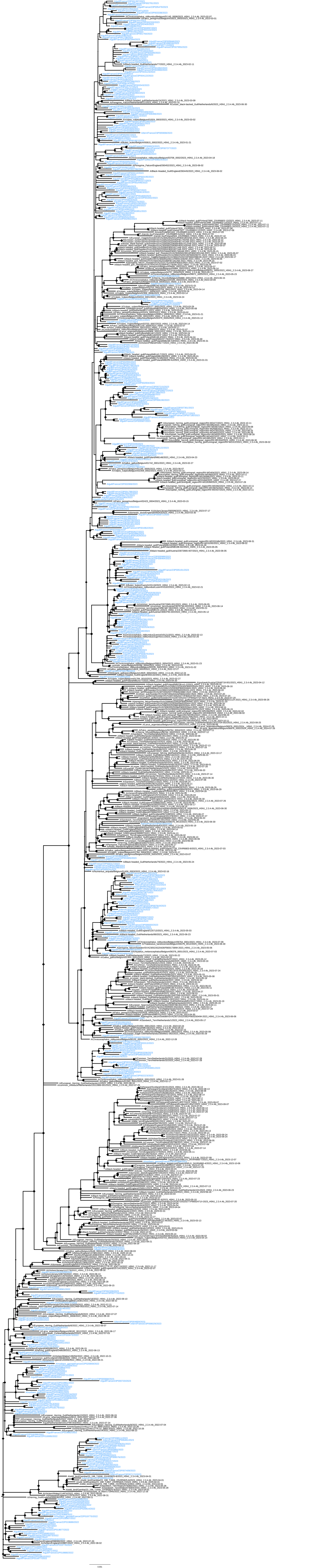

Supplement: Supporting Information 11 — Figure S11: Phylogenetic tree based on the concatenated genome of EA-2022-BB using ML method. [file 8895883.f11.pdf]
